# Research on the factors of extremely short construction period under the sufficient resources based on Grey-DEMATEL-ISM

**DOI:** 10.1371/journal.pone.0265087

**Published:** 2022-03-10

**Authors:** Junlong Peng, Chao Peng, Mengyao Wang, Ke Hu, Dubin Wu

**Affiliations:** 1 College of Transportation Engineering, Changsha University of Science and Technology, Changsha, China; 2 College of Civil and Transportation Engineering, Shenzhen University, Shenzhen, China; Sichuan University, CHINA

## Abstract

Under the condition of sufficient resources, there are many factors affecting the realization of extremely short construction period of engineering construction projects. Based on literature review and questionnaire survey, this paper firstly selected 17 influencing factors from the five dimensions of design, management, technology, policy and environment. And the factor analytic hierarchy process model was established based on Grey-DEMATEL-ISM. The model introduced the improved grey system theory and combined decision-making trial and evaluation laboratory (DEMATEL) with interpretative structural modeling method (ISM). In addition, the model can not only identify the critical factors in the system, but also present the internal logical relationship between the influencing factors through the multi-level hierarchical structure diagram. Finally, through the analysis of the influencing factors of extremely short construction period under the sufficient resources, it defined that the key factor is the natural environment and second is the structure type. The methodology implemented in this paper helps decision makers and managers of construction projects to understand the interrelationship and degree of influence among factors affecting the duration under the condition of sufficient resources, to effectively grasp key factors, and to effectively achieve project success.

## 1. Introduction

Schedule management as an important element of project management, which has attracted the attention of scholars and project managers [1, 2]. At present, the most extensive and in-depth study is the shortest construction period under resource constraints [3–5]. However, with the frequent occurrence of natural disasters and public health events around the world in recent years [6], emergency hospitals, temporary rescue sites and road restoration projects need every builder to make a rapid response to minimize the damage. These projects often have very high requirements for the construction period with little restrictions on resources. Based on this, a new problem is worthy thinking deeply: how to make construction period of the whole project extremely short under the condition of sufficient supply of equipment, raw materials and human resources.

The report titled "Human Costs of Disasters 2000–2019," [7] published by the United Nations, states that the total number of natural disasters worldwide has climbed significantly in the first two decades of the 21st century. In addition, the outbreak of the novel coronavirus (COVID-19) epidemic demonstrates the extreme suddenness of public health events [8]. In the aftermath of these large-scale disasters, projects such as the construction of temporary resettlement buildings, the repair of public infrastructure, and the establishment of emergency hospitals need to be started immediately [9–11]. The completion of each project within a very short construction period has a bearing on the safety of people’s lives, the harmony and stability of society, and the stability of the state regime. In an era of rapid development and change, the study of work schedules under the condition of sufficient resources is of some research significance.

The results of the study can, to a certain extent, guide project leaders in future emergency projects to be able to react quickly, develop reasonable construction plans in a short time, avoid the disadvantages of incomplete consideration due to the short construction time, reducing the waste of public resources in society and achieving maximum social benefits.

To solve this problem, it is first necessary to define the resources and their adequacy [12]. Most of the resource categories are considered from the viewpoint of resource constraints. Basic resource categories are renewable, nonrenewable, and doubly constrained resources [13, 14]. Since the scenarios presented in this paper focus on emergency conditions, a division based on transportability is proposed, which can be divided into transportable and non-transportable resources. Transportable resources are quickly available at every point in the project’s progress. Typically, the majority of resources are transportable and can be replenished as soon as they are consumed during the project. Non-transportable resources are those resources that cannot be successfully replenished under specific circumstances or that take too long to replenish to affect scheduled schedule requirements. According to the actual situation [15], the condition of resource sufficiency mentioned in the article is considered as a situation of resource sufficiency when the number of transportable resources is not less than the demand.

As a whole system, the change of any factor will affect and cause the change of the system [16]. Under the condition of extremely high requirements on the construction period, the location and action path of each factor must be determined. Rash construction will inevitably lead to the delay of the construction period and the failure of the project [17–20]. Therefore, it is very important to analyze the factors affecting the limit of construction period under the condition of sufficient resources and find out the critical factors.

In recent years, many scholars have discussed and studied influencing factors of construction period from different angles and levels. Ephrem et al. [21] divided the influencing factors into success factors and failure factors, and determined the relative importance of these factors by multiple regression analysis. Jeffrey et al. [22] prioritized these reasons according to the importance index of the comprehensive frequency and severity index, and identified five main reasons, including lack of proper planning and scheduling, many change orders from customers. Basem et al. [23] based on a large number of questionnaires, the delay factors are ranked in descending order according to the Relative Importance Index (RII). It is considered that the three factors affecting the construction period of the reconstruction project include site constraints and conditions, electrical and mechanical rerouting works, and design buildability and adjustment. Jawad [24] classified the causes of delay in different stages of the construction project and developed a simplified formula to calculate the impact of various causes of delay on site. Mustafa et al. [25] integrated FCM (the fuzzy cognitive map) method, FDEA (fuzzy data envelopment analysis) method and ISM (interpretive structural modeling) were used to analyze the causal relationship between delay factors in construction projects, and verified the effectiveness of this method with an actual case study in Iran. Basem Al Khatib et al. [26]in reviewing the factors leading to the delay of mataf expansion project, found the existence of other inevitable factors leading to the delay and divided them into demolition stage and construction project. Some studies have also explored the influencing factors of construction period indirectly by using prediction model [27–29], multiple linear regression model [30, 31], smoothing technology [32] and others.

All the above studies have provided important references and suggestions for the exploration of influencing factors of construction period. As we can known, most of the research on the influencing factors of construction period focuses on resource constraints [33–35]. However, in some emergency situations, construction resources are sufficient to ensure rapid construction, so as to avoid huge losses [36–38]. At the same time, in order to help managers to control the construction period, thereby avoiding the shortcomings caused by short time and saving resources. It is necessary to conduct in-depth research on the factors affecting the realization of the extremely short construction period of the construction project under the condition of sufficient resources.

This paper creatively makes a qualitative and quantitative analysis on the internal relationship between the influencing factors of extremely short construction period from the perspective of sufficient resources, and identified the key factors involved. The objectives are: (1) identifying the factors affecting the construction period through a literature review and a questionnaire survey method; (2) Find the key factors among the many influencing factors; and (3) with the help of interpretive structure model (ISM), a multi-level hierarchical structure model is constructed to determine the hierarchical structure of various factors and intuitively display the action relationship between influencing factors. This study not only summarizes and organizes the factors influencing the duration under the condition of sufficient resources, but also identifies the key factors and the internal logical relationships among them based on the con-structed model.

## 2. Factor selection

### 2.1 Extraction of influencing factors based on literature review method

Keywords such as influencing factors of construction period, construction period and delay factors are searched in web of science, Google academic, CNKI and other databases. We selected the relevant literature in recent ten years and made statistics from the five dimensions of design, management, technology, policy and environment, so as to remove the factors that appear too few times. The influencing factors of construction period are preliminarily sorted out. However, there are still some subordinate and inclusive relationships among these factors, which need to be further sorted and classified. In order to ensure the scientific rationality of factor induction, the authors organized a research group, whose members are graduate students in engineering management in universities. The research group studied and discussed the influencing factors of induction. At the same time, some factors with strong inclusion and subordination were reorganized are combined into a capital chain, the lack of skilled workers is included in the shortage of human resources. Finally, 24 independent influencing factors were determined.

### 2.2 Determination of influencing factors based on questionnaire method

Since the premise of the study in this paper is a resource sufficiency condition, the factors affecting the schedule due to the constraint of resource limitation must be excluded. Therefore, the influencing factors must be further screened and identified. In this paper, a questionnaire is drafted by means of a questionnaire survey. The content of the questionnaire is divided into three parts. The first part is the basic information background of the respondents. Because the respondents’ working years, education level and work unit directly affect their understanding of the factors affecting the construction period, it is necessary to clarify the basic information of the respondents. The second part is the questionnaire of influencing factors of construction period, which requires it to confirm and identify the influencing factors under the condition of resource sufficiency, in the form of the Likert level five scale. The respondents are required to score according to the importance of 1 ~ 5. The third part is the subjective question, which is used to collect the respondents’ other opinions on the influencing factors.

A total of 160 questionnaires were distributed and 123 were recovered, of which 98 were valid, and the effective recovery rate was 61.25%. SPSS 25.0 was used to calculate the reliability coefficient of the data to ensure the reliability of the questionnaire. The test results are shown in **Table 1**. The reliability coefficient α = 0.763>0.7, which indicates that it meets the requirements of reliability test and the reliability of the questionnaire is high. After that, the whole sample is statistically analyzed to calculate the sample mean and standard deviation of each factor. The mean value represents the overall evaluation of the importance of factors by respondents in the questionnaire. And the standard deviation indicates the consistency of different respondents’ views on factors. The smaller the standard deviation, the better the consistency. The 25 factors are sorted according to the mean value of the factors, and **Table 2** is obtained. It can be seen from the table that some factors have little impact on the construction period under the condition of sufficient resource and should be screened out. Therefore, the average value of 3 is considered as the benchmark for factor screening, and the factors lower than 3 points are eliminated, and finally 17 influencing factors are obtained. In order to comprehensively analyze the different properties of factors, 17 factors are divided into five categories: project itself, management, logic, environment and organization. The specific factors and their classification are shown in **Table 3**.

**Table 1:** The reliability statistics.

Reliability Statistics		
Cronbach’s Alpha	Cronbach’s Alpha based on standardization term	Number of items
0.763	0.796	25

**Table 2:** The average value of the factors.

influencing factors	Average
Construction safety organization	3.867
Contractor management level	3.714
Maximum construction work surface	3.622
The management level of the owner	3.612
Natural environment	3.592
Competence level of consultants	3.551
Designer’s capability level	3.520
Construction technology	3.500
Social environment	3.469
Articulation of materials or devices	3.408
The connection of construction process steps	3.408
Management level of the supplier	3.378
Political environment	3.286
Total number of floors	3.082
Function	3.051
Structure type	3.010
Total floor area	3.008
Engineering construction standards	2.969
Labor disputes and strikes	2.082
Estimated construction cost	1.582
Equipment	0.541
Material	0.531
Financial chain	0.520
Labor	0.520

**Table 3:** Influencing factors of the minimum construction period under sufficient resources.

Constraint Type	influencing factors	Previous literature
project itself	Total floor area (S1)	[39, 40]
	Total number of floors (S2)	[39, 40]
	Function (S3)	[32]
	Structure type (S4)	[32]
management	The management level of the owner (S5)	[41–43]
	Contractor management level (S6)	[17, 22, 24, 42, 44–47]
	Designer’s capability level (S7)	[22, 24, 41, 44–46]
	Competence level of consultants (S8)	[24, 32, 42, 46]
	Management level of the supplier (S9)	[42, 46, 48]
logic	Construction technology (S10)	[24, 41, 49]
	Maximum construction work surface (S11)	[50, 51]
environment	Political environment (S12)	[24, 41, 42, 52]
	Natural environment (S13)	[32, 44, 52]
	Social environment (S14)	[22, 24, 42]
organization	Articulation of materials or devices (S15)	[22, 24, 41, 42, 44, 46]
	The connection of construction process steps (S16)	[22, 24, 42, 44, 46, 53]
	Construction safety organization (S17)	[41, 43, 54]

### 2.3 Explanation of influencing factors

1. Total floor area(S1): It is the sum of the floor area of single or multiple buildings above and below the ground level within the construction site.
2. Total number of floors(S2): It is the sum of the number of strata and the number of strata above the ground.
3. Function(S3): To meet the specific purpose and use requirements, it includes space composition, functional partitioning, human flow organization, evacuation, etc.
4. Structure type(S4): It refers to both the load-bearing structure and the enclosure structure of its building. The durability, seismic resistance, safety, and space use performance of houses of various structures are different.
5. The management level of the owner(S5): In the process of project construction, the embodiment of the owner’s management ability depends mainly on the size, quality, and structure of the owner’s team.
6. Contractor management level(S6): In the project construction process, the contractor’s management capability is reflected mainly in the size, quality, and structure of the contractor’s management team.
7. Designer’s capability level(S7): The degree of mastery and experience of the designer’s expertise in the field of architectural design.
8. Competence level of consultants(S8): In the process of project construction, the manifestation of the consultant’s management ability depends mainly on the size, quality and structure of the consultant’s management team.
9. Management level of the supplier(S9): In the process of project construction, the embodiment of the supplier’s management ability mainly depends on the scale, quality and structure of the supplier’s management team.
10. Construction technology(S10): Construction techniques for each major type of work in the construction of building projects.
11. Maximum construction work surface(S11): It refers to a certain floor, part, or location on the construction object where workers may be arranged and machinery arranged and is used to reflect the maximum possibility of the construction process to arrange production elements in space.
12. Political environment(S12): It refers to the external political situation of the engineering construction project, the national policy, and its changes.
13. Natural environment(S13): It refers to the environment formed by natural things such as water, soil, region, and climate.
14. Social environment(S14): It refers to the network of relationships between the organization and various publics, including collaborative relationships between parties, coordination of relationships with governmental publics.
15. Articulation of materials or devices(S15): When a process starts during construction, the corresponding materials or equipment can be in place, i.e., the coordination of equipment or materials.
16. The connection of construction process steps(S16): After the end of a certain process in the construction process, the latter process can follow in time, i.e., the coordination of the various steps and processes of construction.
17. Construction safety organization(S17): It covers all security aspects of the operation and involves management, finance, and logistics.

## 3. Methodology

The essence of influencing factor research is to identify the key factors and clarify the action ways between the factors. There are many kinds of research methods, such as PSR theoretical framework method [55], data envelopment analysis method [56], ISM [25, 57] and others. PSR theoretical framework method as a qualitative method, is less objective. Data envelopment analysis can appropriately express the connotation of the main factors, but it requires a high number of samples. ISM can effectively decompose complex systems without quantitative supplement. Facing these problems, this paper proposes to integrate decision experiment and evaluation laboratory method (DEMATEL) with interpretative structure model (ISM). DEMATEL constructs complex causality with the help of matrix or directed graph, and describes its relationship in detail [58]. ISM can model the structure of complex systems, and the combination of the two can overcome the shortcomings when they are used alone. Therefore, the integrated DEMATEL-ISM method is widely used in various fields [59]. The context of this study is the resource adequacy condition. There have in fact been relevant cases, but the problem of insufficient data remains. To deal with this problem, this paper considers the use of grey theory [60] to address the problem of insufficient or incomplete data, used in conjunction with DEMATEL-ISM, to provide a new approach to dealing with inter-factor relationship studies with fewer cases.

### 3.1 The process of model establishment

**Fig 1:**
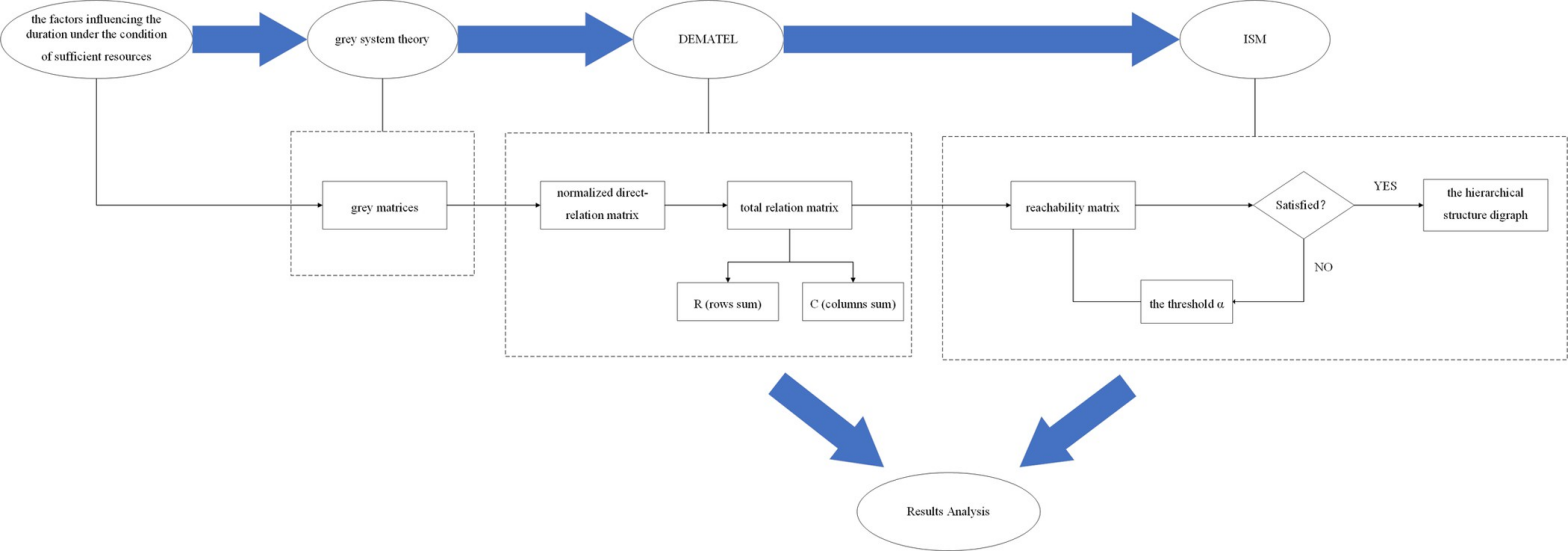
The process of Grey-DEMATEL-ISM analysis model.

DEMATEL and ISM are combined to analyze the influencing factors and improving them with gray theory. The duration analysis model of Grey-DEMATEL-ISM is constructed, and the specific process is shown in **Fig 1**. Firstly, the main factors affecting the realization of very short construction period under the condition of sufficient resources are obtained. Then, the grey system theory is used to improve the expert scoring results and construct the grey number matrix. Secondly, the influencing factors are analyzed by DEMATEL. In this analysis process, the first step is to standardize and clarify the gray matrix obtained earlier by using CFCs method, and take the weight of experts into account to construct a direct impact matrix; The second step is to normalize the direct influence matrix, and consider the indirect relationship of each factor to obtain the comprehensive influence matrix, so as to determine the influence degree and affected degree of each influencing factor. Finally, the hierarchical structure diagram is constructed by ISM, and the comprehensive influence matrix is transformed into reachable matrix in MATLAB environment. After that, the threshold α is introduced to remove the redundant information in the reachability matrix. The simplified reachability matrix is divided into influencing factor levels to visually display the action relationship between influencing factors.

### 3.2 Modeling process

Step 1 Expert Interview Design.

Invite 2 ~ 6 experts [61] to compare the influence relationship between row factors and column factors in the matrix according to their own experience (**Table 4**) and expert semantic variable table (**Table 5**).

**Table 4:** Expert semantic variables.

Numerical value	Definition
0	No impact
1	Light impact
2	Medium impact
3	Height Impact
4	Extremely high impact

**Table 5:** Semantic variables of expert weights.

Semantic variables	Weighted Gray Number
Not important	[0, 0.3]
Less important	[0.3, 0.5]
important	[0.4, 0.7]
More important	[0.5, 0.9]
Very important	[0.7, 1]

Step 2 Develop specific grey matrices “B”.

The direct influence matrix obtained by expert scoring is transformed into grey number matrix B by using interval grey number. This is, according to the expert evaluation grey number semantic variable table (**Table 6**), the value scored by the expert is transformed into the corresponding interval grey number, in which the interval grey numbers on the diagonal are [0,0].

**Table 6:** Semantic variables of the expert evaluation gray numbers.

Numerical value	Gray Number	Definition
0	[0, 0]	No impact
1	[0, 0.25]	Light impact
2	[0.25, 0.5]	Medium impact
3	[0.5, 0.75]	Height Impact
4	[0.75, 1]	Extremely high impact

Step 3 Convert average grey matrix into crisp relationship matrix.

The Gray number matrix B obtained in step 2 is normalized according to Eq (1), and then clarified according to Eq (2) to derive the direct influence matrix Z_k_ for each expert clarity value, k is the number of expert numbers. Similarly, the given expert weights are clarified to derive the specific expert weights W_k_, k is the number of expert numbers.


$$\{\begin{array}{l} \bar{⊗}X_{ij}^{k}=(\bar{⊗}X_{ij}^{k}-\min\bar{⊗}X_{ij}^{k})/{▲}_{\min}^{\max}\\ \underline{⊗}X_{ij}^{k}=(\underline{⊗}X_{ij}^{k}-\min\underline{⊗}X_{ij}^{k})/{▲}_{\min}^{\max}\\ \;{▲}_{\min}^{\max}=\max\bar{⊗}X_{ij}^{k}-\min\underline{⊗}X_{ij}^{k} \end{array}$$ (1)

$$\{\begin{array}{c} Y_{ij}^{k}=\frac{\left[\underline{⊗}X_{ij}^{k}(1-\underline{⊗}X_{ij}^{k})+(\bar{⊗}X_{ij}^{k}\times \bar{⊗}X_{ij}^{k})\right]}{1-\underline{⊗}X_{ij}^{k}+\bar{⊗}X_{ij}^{k}}\\ Z_{ij}^{k}=\min\bar{⊗}X_{ij}^{k}+Y_{ij}^{k}\times {▲}_{\min}^{\max} \end{array}$$ (2)

Where, $\bar{⊗}X_{ij}^{k}、\underline{⊗}X_{ij}^{k}$ are the upper and lower bounds of expert evaluation respectively.

Step 4 Determine Direct Influence Matrix “M” through Eq (3) and Eq (4).

$$\left\{ \begin{array}{c} Z_{ij}=W_{1}Z_{ij}^{1}+W_{2}Z_{ij}^{2}+\cdots +W_{k}Z_{ij}^{k}\\ \sum_{k=1}^{n}W_{k}=1 \end{array}\right.$$ (3)

$$\left\{ \begin{array}{c} M=Z/S\\ S=\max\sum_{j=1}^{n}Z_{ij},\ldots ,j=1,2\ldots n \end{array}\right.$$ (4)

Where, w_k_ is the expert weight after clarification, $Z_{ij}^{k}$ is the matrix of the direct influence of the clear value of the kth expert on the influence factor in row i, column j.

Step 5 Obtain “T” (the Total Relation Matrix) through Eq (5).

$$T=M*{\left(I-M\right)}^{-1}$$ (5)

Step 6 Determine causal factors.

Calculate R (rows sum) and C (columns sum) using Eq (6) and (7).


$$R={\left(\sum_{j=1}^{n}m_{ij}\right)}_{n\times 1}$$ (6)

$$C={\left(\sum_{i=1}^{n}m_{ij}\right)}_{1\times n}$$ (7)

Step 7 Preparation of a causal diagram.

Step 8 Select the threshold α. The adjacency matrix *A* is determined by Eq (8).


$$r_{ij}=\left\{ \begin{array}{c} 1,\;z_{ij}\geq \alpha \\ 0,\;z_{ij}<\alpha \end{array}\right.\;Adjacency\;matrixA=(r_{ij}{)}_{n\times n}$$ (8)

Step 9 Develop of reachability matrix.

The matrix *A* should be developed further until it satisfies the conditions of equation where the obtained matrix *R* is reachability matrix. This calculation process can be realized in MATLAB.


$${\left(A+E\right)}^{1}\neq {\left(A+E\right)}^{2}\ldots .\neq {\left(A+E\right)}^{K-1}={\left(A+E\right)}^{K}=M$$ (9)

Where, *E* is an identity matrix.

Step 10 Hierarchy division.

Partition the factors into different levels. According to the reachability matrix *R* and Eqs (10)~(12), the reachable set *A*(*S*_*i*_), the antecedent set *B*(*S*_*j*_) and the intersection set *A*(*S*_*i*_)∩*B*(*S*_*j*_) are obtained. And the set *C*(*S*_*i*_) of all the factors that can reach *S*_*i*_. The highest element is determined by Eq (12). Next, the first level factors will be determined and removed from the matrix. Repeating this method to determine the highest-level feature set of each level, and dividing all factors into corresponding levels. Finally, the hierarchical structure digraph of the influencing factors of the hidden cost of prefabricated buildings is obtained.


$$A(S_{i})=\left\{S_{j}|S_{j}\in S,S_{\mathrm{ij}}=1\right\}$$ (10)

$$B(S_{j})=\left\{S_{i}|S_{i}\in S,S_{\mathrm{ij}}=1\right\}$$ (11)

$$C(S_{i})=\{S_{i}|S_{i}\in S,A(S_{i})\cap B(S_{i})=R(S_{i})\}$$ (12)

## 4. Model application

### 4.1 Application of Grey-DEMATEL-ISM model

With the outbreak of the novel coronavirus (COVID-19) epidemic in Wuhan, China, in January 2020, the escalating number of cases overwhelmed the admission capacity of the designated hospitals. Two specialty field hospitals-Huoshenshan and Leishenshan-were designed, built and commissioned in 9–12 days to address the outbreak [62, 63]. The successful completion of these two specialty field hospitals eventually accelerated the control of the outbreak. The rapid completion of these two hospitals has brought new discussion points to our engineering and construction industry.

A great deal of practical experience exists in the rapid construction industry, but it has not been possible to generalize the theory. Four experts were invited to compare the influence of row factors on column factors, analyze and score them. These four experts gave the corresponding interval gray weights according to their respective characteristics (**Table 7**).

**Table 7:** Expert weights.

Number	Experts	Weighted Gray Number
1	University Professor (Construction Management)	[0.5, 0.9]
2	Construction Project Manager / Senior Engineer	[0.5, 0.9]
3	Consulting Engineer	[0.4, 0.7]
4	Construction Worker	[0.4, 0.7]

The specific scoring tables collected were organized into corresponding scoring matrices, and then the scoring matrices were converted into gray number matrices using interval gray numbers. Based on Eq (1) and Eq (2), the grey matrix and expert weights obtained above are clarified by applying the CFCS method. Furthermore, the direct influence matrix Z (**Table 8**) can be obtained by Eq (3).

**Table 8:** The direct influence matrix Z.

	S1	S2	S3	S4	S5	S6	S7	S8	S9	S10	S11	S12	S13	S14	S15	S16	S17
S1	0.00	0.48	0.16	0.25	0.21	0.41	0.41	0.54	0.04	0.41	0.46	0.00	0.26	0.06	0.10	0.14	0.34
S2	0.46	0.00	0.21	0.63	0.23	0.48	0.55	0.45	0.04	0.47	0.25	0.07	0.32	0.03	0.32	0.36	0.48
S3	0.38	0.32	0.00	0.48	0.20	0.43	0.55	0.18	0.21	0.41	0.25	0.20	0.18	0.15	0.11	0.28	0.13
S4	0.32	0.32	0.55	0.00	0.25	0.70	0.70	0.45	0.49	0.75	0.55	0.21	0.32	0.09	0.45	0.61	0.55
S5	0.15	0.21	0.27	0.27	0.00	0.55	0.48	0.43	0.43	0.20	0.18	0.54	0.06	0.38	0.32	0.05	0.43
S6	0.11	0.03	0.03	0.09	0.13	0.00	0.23	0.30	0.46	0.75	0.55	0.21	0.66	0.25	0.70	0.75	0.75
S7	0.21	0.16	0.11	0.11	0.07	0.52	0.00	0.20	0.09	0.54	0.34	0.04	0.45	0.05	0.09	0.38	0.20
S8	0.09	0.03	0.05	0.05	0.11	0.59	0.33	0.00	0.29	0.45	0.25	0.06	0.27	0.25	0.23	0.38	0.75
S9	0.00	0.00	0.03	0.00	0.09	0.34	0.11	0.13	0.00	0.14	0.03	0.00	0.14	0.09	0.75	0.29	0.47
S10	0.29	0.41	0.54	0.41	0.11	0.59	0.68	0.51	0.45	0.00	0.52	0.04	0.32	0.00	0.46	0.68	0.48
S11	0.18	0.03	0.03	0.11	0.09	0.41	0.47	0.23	0.14	0.52	0.00	0.00	0.27	0.04	0.68	0.51	0.52
S12	0.38	0.38	0.29	0.29	0.32	0.29	0.22	0.29	0.09	0.12	0.09	0.00	0.16	0.54	0.03	0.09	0.68
S13	0.52	0.52	0.34	0.47	0.11	0.41	0.48	0.25	0.28	0.64	0.42	0.21	0.00	0.39	0.25	0.23	0.57
S14	0.16	0.16	0.41	0.32	0.40	0.23	0.16	0.25	0.16	0.09	0.03	0.68	0.15	0.00	0.03	0.09	0.68
S15	0.03	0.00	0.03	0.11	0.05	0.43	0.10	0.36	0.64	0.45	0.52	0.04	0.39	0.00	0.00	0.57	0.59
S16	0.03	0.00	0.03	0.09	0.09	0.54	0.29	0.32	0.20	0.64	0.34	0.00	0.30	0.03	0.54	0.00	0.57
S17	0.14	0.29	0.14	0.16	0.36	0.64	0.55	0.63	0.11	0.70	0.39	0.20	0.27	0.43	0.38	0.45	0.00

The direct influence matrix Z is normalized according to Eq (4). On the basis of considering the indirect relationship of various factors, the total relationship matrix T (**Table 9**) by using Eq (5).

**Table 9:** The total relation matrix T.

	S1	S2	S3	S4	S5	S6	S7	S8	S9	S10	S11	S12	S13	S14	S15	S16	S17
S1	0.36	0.80	0.48	0.63	0.48	1.25	1.10	1.14	0.51	1.27	1.08	0.23	0.81	0.35	0.76	0.88	1.21
S2	0.89	0.44	0.63	1.07	0.58	1.54	1.42	1.22	0.65	1.56	1.06	0.35	1.01	0.39	1.13	1.28	1.56
S3	0.73	0.67	0.35	0.86	0.49	1.28	1.25	0.81	0.69	1.28	0.89	0.42	0.75	0.43	0.78	1.02	1.03
S4	0.87	0.85	1.04	0.61	0.69	2.03	1.78	1.42	1.25	2.11	1.55	0.56	1.21	0.54	1.52	1.78	1.94
S5	0.53	0.57	0.61	0.66	0.33	1.43	1.18	1.07	0.93	1.10	0.83	0.80	0.66	0.71	1.01	0.84	1.39
S6	0.55	0.48	0.46	0.58	0.50	1.14	1.14	1.13	1.11	1.86	1.37	0.50	1.36	0.63	1.58	1.70	1.92
S7	0.51	0.45	0.38	0.43	0.30	1.20	0.60	0.72	0.50	1.26	0.88	0.22	0.91	0.29	0.68	0.99	0.95
S8	0.40	0.35	0.35	0.40	0.38	1.37	0.97	0.60	0.74	1.27	0.85	0.29	0.81	0.53	0.89	1.08	1.57
S9	0.18	0.18	0.19	0.20	0.25	0.83	0.48	0.50	0.32	0.67	0.42	0.14	0.48	0.26	1.13	0.74	0.99
S10	0.76	0.85	0.94	0.92	0.50	1.78	1.63	1.36	1.11	1.27	1.40	0.33	1.12	0.40	1.42	1.71	1.71
S11	0.48	0.34	0.32	0.45	0.35	1.22	1.10	0.83	0.63	1.35	0.64	0.20	0.82	0.30	1.31	1.22	1.35
S12	0.73	0.73	0.62	0.68	0.63	1.11	0.91	0.91	0.53	0.95	0.68	0.28	0.68	0.83	0.63	0.76	1.51
S13	1.00	0.99	0.80	1.00	0.52	1.57	1.45	1.12	0.92	1.80	1.28	0.53	0.77	0.77	1.15	1.23	1.76
S14	0.50	0.51	0.71	0.67	0.69	1.00	0.81	0.82	0.57	0.85	0.57	0.91	0.62	0.33	0.59	0.71	1.45
S15	0.33	0.30	0.31	0.43	0.32	1.22	0.75	0.93	1.09	1.27	1.10	0.23	0.91	0.28	0.72	1.27	1.43
S16	0.33	0.31	0.32	0.42	0.34	1.31	0.92	0.89	0.68	1.44	0.94	0.20	0.83	0.30	1.17	0.73	1.38
S17	0.58	0.72	0.56	0.65	0.72	1.71	1.42	1.40	0.75	1.78	1.20	0.51	1.00	0.79	1.23	1.39	1.19

R+C and R-C (**Table 10**) can be calculated based on the integrated influence matrix T (Table 9). Where, the row sum (R) indicates the influence degree of the influence factor, the column sum (C) indicates the influenced degree of the influence factor.

**Table 10:** R+C and R-C.

Si	R	C	R+C	R-C
S1	1.82	1.33	3.16	0.49
S2	2.30	1.31	3.60	0.99
S3	1.88	1.24	3.12	0.64
S4	2.98	1.46	4.44	1.51
S5	2.01	1.11	3.11	0.90
S6	2.46	3.14	5.61	-0.68
S7	1.54	2.59	4.13	-1.04
S8	1.76	2.31	4.07	-0.55
S9	1.09	1.78	2.87	-0.69
S10	2.63	3.16	5.79	-0.53
S11	1.76	2.29	4.05	-0.52
S12	1.80	0.92	2.72	0.88
S13	2.55	2.02	4.58	0.53
S14	1.69	1.11	2.80	0.57
S15	1.77	2.42	4.19	-0.66
S16	1.71	2.64	4.36	-0.93
S17	2.41	3.33	5.74	-0.92

**Fig 2:**
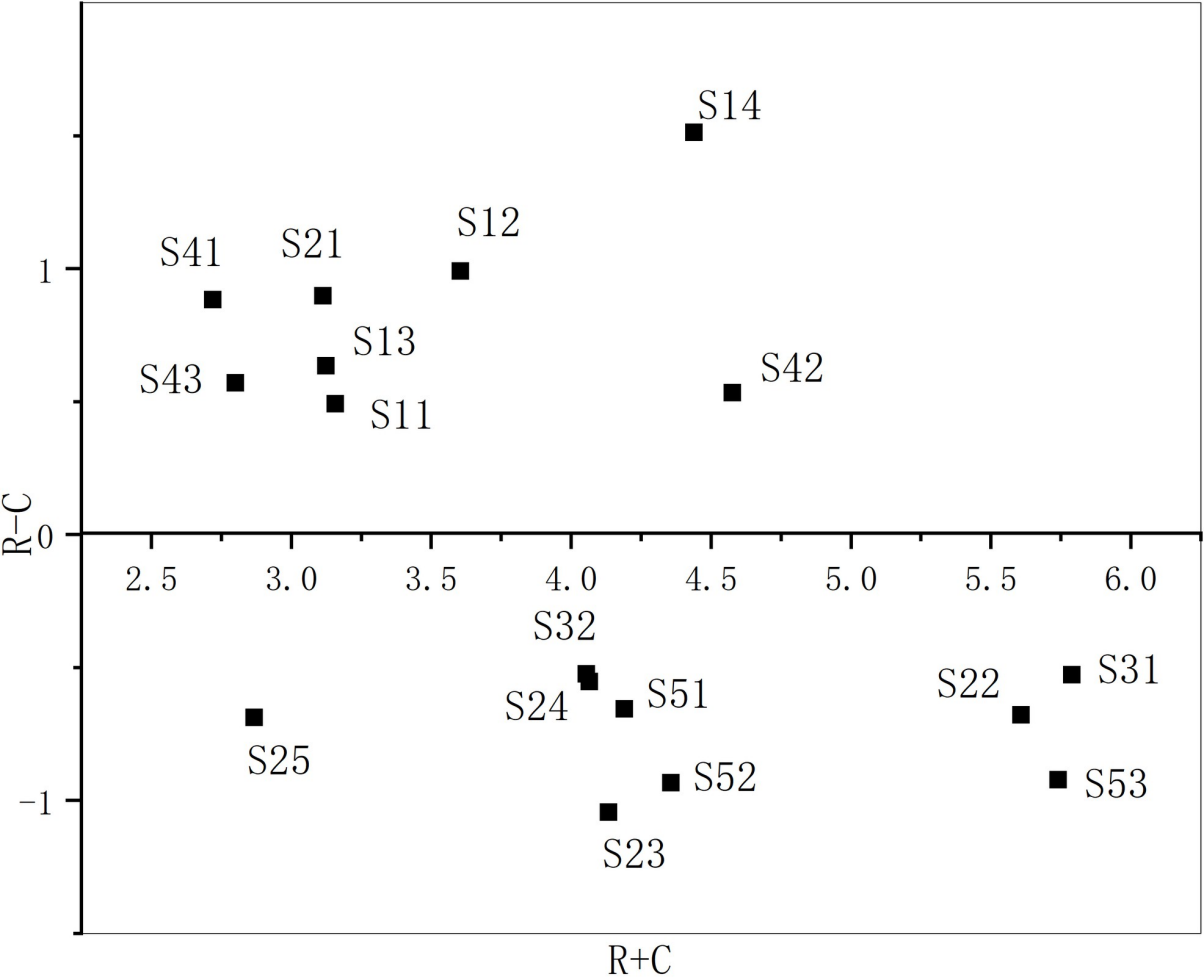
The Cartesian coordinate system of R+C and R-C.

In order to visualize R+C and R-C, a Cartesian coordinate system is drawn based on the relevant indicators to form a scatter plot (**Fig 2**).

After analyzing the attenuation of node degree, set the threshold of comprehensive matrix α = 0.12. According to the comprehensive influence matrix, the reachability matrix R (**Table 11**) can be calculated.

**Table 11:** The reachability matrix R.

	S1	S2	S3	S4	S5	S6	S7	S8	S9	S10	S11	S12	S13	S14	S15	S16	S17
S1	1	0	0	0	0	1	1	1	0	1	1	0	0	0	0	1	1
S2	1	1	0	1	0	1	1	1	0	1	1	0	1	0	1	1	1
S3	0	0	1	0	0	1	1	0	0	1	1	0	0	0	0	1	1
S4	0	0	1	1	0	1	1	1	1	1	1	0	1	0	1	1	1
S5	0	0	0	0	1	1	1	1	1	1	0	0	0	0	1	0	1
S6	0	0	0	0	0	1	1	1	1	1	1	0	1	0	1	1	1
S7	0	0	0	0	0	1	1	0	0	1	0	0	1	0	0	1	1
S8	0	0	0	0	0	1	1	1	0	1	0	1	0	0	1	1	1
S9	0	0	0	0	0	0	0	0	1	0	0	0	0	0	1	0	1
S10	0	0	1	1	0	1	1	1	1	1	1	0	1	0	1	1	1
S11	0	0	0	0	0	1	1	0	0	1	1	0	0	0	1	1	1
S12	0	0	0	0	0	1	1	1	0	1	0	1	0	0	0	0	1
S13	1	1	0	1	0	1	1	1	1	1	1	0	1	0	1	1	1
S14	0	0	0	0	0	1	0	0	0	0	0	1	0	1	0	0	1
S15	0	0	0	0	0	1	0	1	1	1	1	0	1	0	1	1	1
S16	0	0	0	0	0	1	1	1	0	1	1	0	0	0	1	1	1
S17	0	0	0	0	0	1	1	1	0	1	1	0	1	0	1	1	1

**Fig 3:**
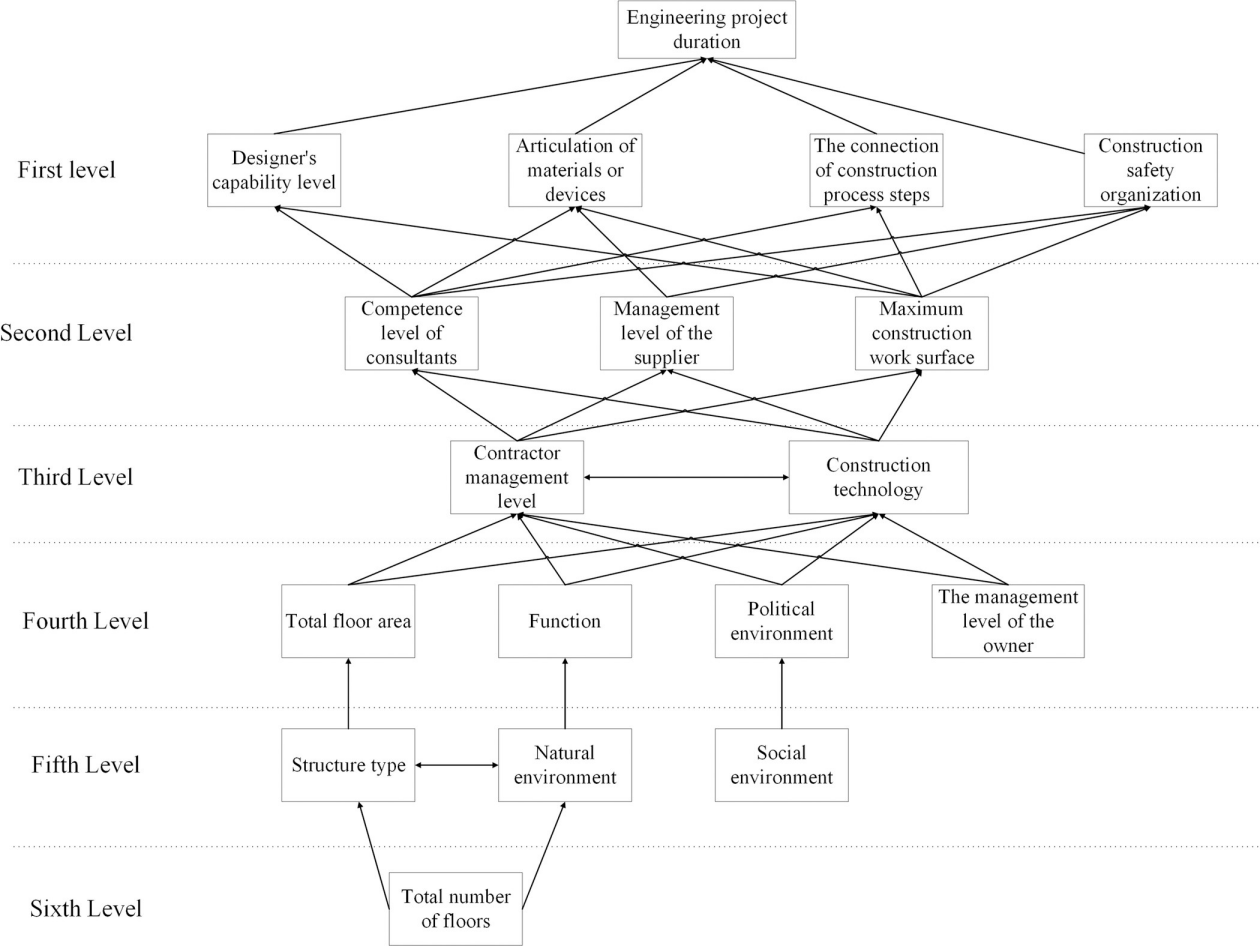
The hierarchical structure digraph of the influencing factors of the construction period.

The reachability matrix R is hierarchically divided by Eq (12) to obtain the corresponding multi-level hierarchical structure model of factors affecting extremely short construction period (**Fig 3**).

### 4.2 Sensitivity analysis

**Fig 4:**
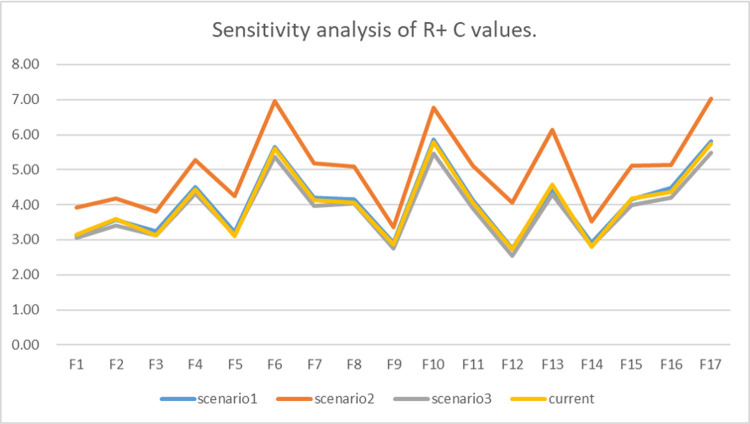
Sensitivity analysis of R+ C values.

**Fig 5:**
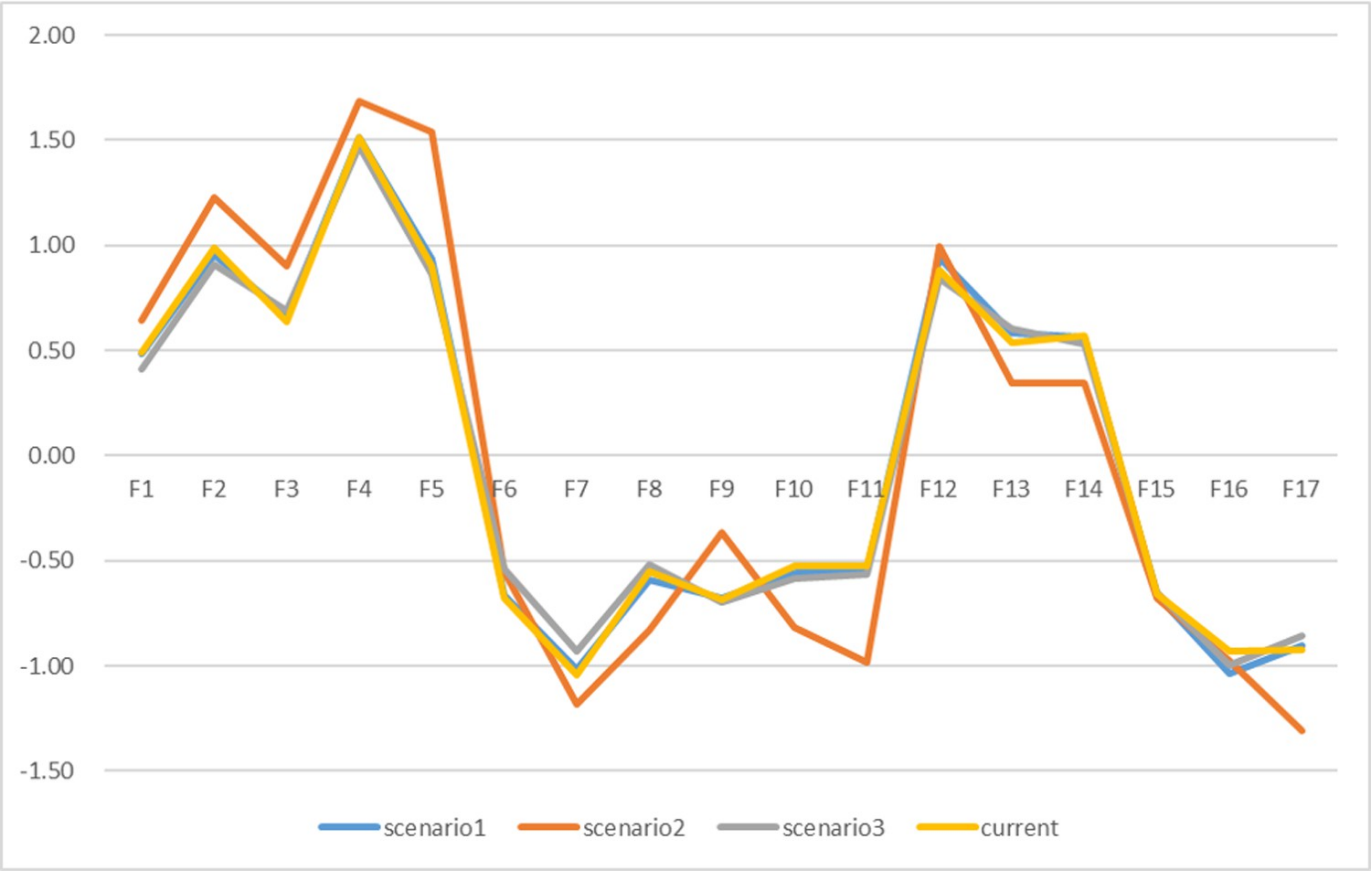
Sensitivity analysis of R-C values.

Sensitivity analysis aims to verify the stability of the results by redistributing the weights of experts [64]. **Table 12** shows the weight redistribution of experts. Referring to previous studies, we analyzed R + C and R-C of influencing factors in different scenarios. **Figs 4** and **5** show the results respectively. In addition, this paper conducted further research on the ISM model (**Table 13**) and found that there are certain fluctuations among the hierarchical factors, but only limited to the changes between the two levels. Most of the influencing factors are not affected by the expert weight, and the overall relationship remained the same. Therefore, there is stability and consistency in relationship evaluation.

**Table 12:** Weight assignment for respondents of sensitivity analysis.

	scenario1	scenario2	scenario3	current
Expert1	[0.5, 0.9]	[0.5, 0.9]	[0.5, 0.9]	[0.5, 0.9]
Expert2	[0.5, 0.9]	[0.5, 0.9]	[0.5, 0.9]	[0.5, 0.9]
Expert3	[0.5, 0.9]	[0.4, 0.7]	[0.5, 0.9]	[0.4, 0.7]
Expert4	[0.4, 0.7]	[0.5, 0.9]	[0.5, 0.9]	[0.4, 0.7]

**Table 13:** Sensitivity analysis of ISM levels.

Level	scenario1	scenario2	scenario3	current
S1	4	3	4	4
S2	5	4	5	6
S3	2	3	4	4
S4	4	6	4	5
S5	4	6	4	4
S6	1	2	3	3
S7	1	1	2	1
S8	3	1	3	2
S9	2	2	2	2
S10	3	1	3	3
S11	1	1	1	2
S12	4	5	4	4
S13	5	3	5	5
S14	5	2	5	5
S15	1	1	1	1
S16	1	1	1	1
S17	1	1	1	1

### 4.3 Analysis of results

**Fig 2** shows that, the 17 influencing factors are divided into two groups. Among them, S1, S2, S3, S4, S5, S12, S13 and S14 are positive values, which are divided into cause factor groups; S6, S7, S8, S9, S10, S11, S15, S16 and S17 are negative values, which are divided into result factor groups.

1. The cause group: causal factors refer to factors with a causal degree R-C> 0. They are S4, S2, S5, S12, S3, S14, S13 and S1 in order. The higher the ranking, the greater the impact of this factor on other influencing factors. The structural type (S4) ranks first because its influence degree (R) is very large. Consequently, its influence degree (C) is low. Generally, only when the structure type is determined can the corresponding schedule be developed, but there exists a choice to change the structure type of a project in order to shorten the schedule [1].
2. The result group: result factors refer to the factor with cause degree R-C< 0, which is sorted according to the absolute value, followed by S7, S16, S17, S9, S6, S15, S8, S10 and S11. The higher the ranking, the greater the degree of influence of other factors. The reason why management level of the designer (S7) ranks first is that its influence degree is too low, but it is highly influenced by other factors. Similar to this factor, it shows a strong passivity and is easy to be affected by other influencing factors, which further affects the construction period.

The R+C of each influencing factor in the system represents the importance of the influencing factor in the system to a certain extent. The cause group are taken as the key factors, and the order from large to small is S13, S4, S2, S1, S3, S5, S14 and S12. The results demonstrate that the natural environment (S13) is the most critical factor affecting the construction period, and second is the structure type (S4).

As can be seen in **Fig 3**, all influencing factors are divided into three orders and six layers. The factors located in the first and second layers are called direct influencing factors, this layer is a direct factor affecting the construction period. The third and fourth layers are intermediate influencing factors, which play the role of intermediate transition. The factors on the fifth and sixth layers are called fundamental factors, which play a decisive role in the construction period. The fundamental factors include S2(total number of floors), S4(structure type), S13(natural environment), S14(social environment).

## 5. Discussion

Based on the literature review and questionnaire survey, this paper identified 17 factors affecting the realization of extremely short duration under resource-sufficient conditions. Through the analysis of the influencing factors by Grey-DEMATEL-ISM model, it was found that the impact of each constraint dimension on the extremely short construction period is mainly reflected in the complex relationship among the influencing factors. Among them, the natural environment is the central factor, which plays a key role in the whole influencing factor system. In addition, the multi-level hierarchical structure model of factors we proposed divided all influencing factors into three categories: direct influencing factors, intermediate influencing factors and fundamental influencing factors. It can determine the hierarchical structure of each factor, and visually display the action relationship between influencing factors. Therefore, the model can not only effectively identify and analyze the key factors affecting the realization of extremely short construction period under the condition of sufficient resources, but also directly reflect the complex internal relationship between various factors through the hierarchy diagram.

The key factors obtained by the DEMATEL model coincide with the fundamental factors obtained by the ISM, which fully demonstrates the importance of the natural environment as an influencing factor. At present, there is evidence that the ecological environment occupies an important position in the construction of the whole engineering project [32, 44, 52], especially as the government’s attention to the ecological environment increases year by year [65, 66], achieving rapid construction and building without damaging the ecological environment has become one of the research directions in the new situation in recent years. In addition, the type of structure and the total number of floors have a great influence in achieving a fast construction process. The type of structure of the building includes masonry-concrete structure, reinforced concrete structure, steel structure, etc [67]. After classifying the structural types of different buildings, Wang PP [68] found that the project duration was more significantly affected by different structural types, and Hu Wenfa [40] also considered the influence of structural types in the duration prediction model. The total number of stories as a commonly used parameter in duration prediction models [40] reflects the fact that the number of building stories is a critical aspect in determining duration, and there are many studies [69–71] related to high-rise construction.

This study innovatively investigates the influencing factors of extremely short construction period under the sufficient resource. The formulated Grey-DEMATEL-ISM mode studies the relationship between various factors, and obtains its critical factors. The results provide a certain theoretical basis for the realization of extremely short duration under the condition of sufficient resources, and broaden the ideas for the follow-up research on the extremely short construction period.

Despite the significance of these outcomes, the present work still has some limitations. Although the DEMATEL-ISM method has been improved using gray systems theory, the model remains extremely dependent on the judgment of a panel of experts [72]. In addition, the factors identified in this study were limited to housing construction projects, but there are many types of construction projects and each has its own unique factors. The factors in this paper are mainly drawn from the relevant literature of the last decade, with some external factors not taken into account, such as building information modelling modular construction and 5G communications [63]. These issues should be considered and addressed in future research. And the researchers recommend the use of exact statistics from different accident analyses, combined with a dynamic approach, to further investigate the factors that influence the achievement of very short durations.

## 6. Conclusion

This paper innovatively proposes to study the schedule problem under the sufficient resources and investigates the limiting factors that affect the achievement of very short schedules. Various factors were extracted from the known literature, relied on the experience of relevant practitioners to filter and summarize them, and were analyzed using a hybrid Multi-Criteria Decision-Making approach of Grey-DEMATEL and ISM. The results of the two methods respond to each other, the analyses reveal that the natural environment is the most critical factor affecting the achievement of very short construction period under the condition of resource tolerance, and decision makers and constructors should grasp this critical factor to prepare for the smooth execution of the project under special circumstances.

The contribution of the study is twofold. Firstly, it identifies the factors affecting the construction schedule based on the previous work by Delphi method and questionnaire survey, and determines the critical factors. Secondly, the study proposed the Grey-DEMATEL-ISM model to study the interactions between factors, providing decision makers with many insights and guiding ideas to identify critical issues in order to avoid affecting the construction schedule.

This study provides several directions for future research. Since the model proposed in this study only examines the duration domain, but the model itself is used to study the relationship between factors, the same model can be applied to the study between other factors. In addition, the basis of this study lies in the condition of sufficient resources, which is an engineering problem in special cases. In previous studies, examples of special cases are generally seldom considered as too extreme, but with the development of society, the possibility of such polarized cases appears more and more, for which we must study in advance and prepare theoretically.

## Supporting information

S1 File Questionnaire file. (PDF)

S2 File The basic information of the respondents file. (XLSX)

S3 File The list of research groups file. (XLSX)

S4 File Interview consent file. (DOCX)

S5 File Expert information file. (XLSX)

S6 File Interview process file. (PDF)

S7 File Interview results file. (XLSX)
