# Supplementary material for: Research on the factors of extremely short construction period under the sufficient resources based on Grey-DEMATEL-ISM
Source: PLoS One. 2022 Mar 10;17(3):e0265087. doi: 10.1371/journal.pone.0265087 (PMC8912191; doi:10.1371/journal.pone.0265087)
Supplement: S1 File — (PDF) [file pone.0265087.s001.pdf]

Questionnaire Survey on Factors Affecting Short Duration under Sufficient Resources

Thank you very much for taking the time out of your busy schedule to fill out this questionnaire! The purpose of this survey is to provide valuable data for "determining the factors influencing the achievement of very short construction periods under adequate resource conditions", and we hope you will answer truthfully with reference to relevant information and your own work experience. **All data results of this questionnaire will be used for academic research only. Please complete this questionnaire if you agree to authorize it.**

Thank you for your help and support! We wish you a successful career and good health!

Part1.The basic information

1. Nature of work unit

A . Engineering Design Institute
B . Construction Company
C . Engineering consulting firm
D . Scientific Research Institutes
E . Other

2. Working years engaged in the engineering field

A . Less than five years
B . Between five to ten years
C . Between ten to twenty years
D . More than twenty years

3. Educational background

A. Below bachelor degree
B. Bachelor degree
C. Master degree
D. Doctor degree or above

4. Professional ranks and titles

A. Primary title
B. Middle title
C. Vice- senior title
D. Senior title

Part2. Investigation on the importance of influencing factors of construction period under Sufficient Resources

The second part is the questionnaire of influencing factors of construction period, which requires it to confirm and identify the influencing factors **under the condition of resource sufficiency**, in the form of the Likert level five scale. The respondents are required to score according to the importance of 1 ~ 5(The scoring rules are as follows:1-Unimportant;2- Generally important;3- Relatively important;4- Important;5- Very important).

**Influencing factors**	**1**	**2**	**3**	**4**	**5**
Financial chain	○	○	○	○	○
Labor	○	○	○	○	○
Material	○	○	○	○	○
Equipment	○	○	○	○	○
The management level of the owner	○	○	○	○	○
Contractor management level	○	○	○	○	○
Designer's capability level	○	○	○	○	○
Competence level of consultants	○	○	○	○	○
Management level of the supplier	○	○	○	○	○
Construction technology, method and process	○	○	○	○	○
Maximum construction work surface	○	○	○	○	○
Political environment	○	○	○	○	○
Natural environment	○	○	○	○	○
Social environment	○	○	○	○	○
Articulation of materials or devices	○	○	○	○	○
The connection of construction process steps	○	○	○	○	○

Labor disputes and strikes	○	○	○	○	○
Total floor area	○	○	○	○	○
Total number of floors	○	○	○	○	○
Function	○	○	○	○	○
Structure type	○	○	○	○	○
Engineering construction standards	○	○	○	○	○
Estimated construction cost	○	○	○	○	○
Construction safety organization	○	○	○	○	○

Part3. Opinions and suggestions

The third part is the subjective question, which is used to collect the respondents' other opinions on the influencing factors.

Your opinion on this questionnaire survey:______________________________________
____

Your suggestion on this questionnaire survey:____________________________________
____
