# Supplementary material for: Research on the factors of extremely short construction period under the sufficient resources based on Grey-DEMATEL-ISM
Source: PLoS One. 2022 Mar 10;17(3):e0265087. doi: 10.1371/journal.pone.0265087 (PMC8912191; doi:10.1371/journal.pone.0265087)
Supplement: S4 File — (DOCX) [file pone.0265087.s004.docx]

Interview Consent

Dear Sir/Madam:

After the outbreak of COVID-19, the rapid establishment of Fire God Mountain hospital and Thunder God Mountain hospital raised new thoughts in the field of engineering management.

Under the condition of sufficient resources, how to complete the engineering and construction project efficiently and quickly while ensuring quality. Although the resource constraints are relatively small, there is still the problem of constraints on the duration. This group hopes to analyze the relationship between factors affecting the duration from this perspective and explore how to control these factors to achieve the extremely short duration.

We guarantee that the interview results and all relevant information obtained in the interview will only be used in academic research. The relevant information of the interviewee is strictly confidential.

Thank you very much for your support!

Research Group on extremely short construction period
