# Supplementary material for: Research on the factors of extremely short construction period under the sufficient resources based on Grey-DEMATEL-ISM
Source: PLoS One. 2022 Mar 10;17(3):e0265087. doi: 10.1371/journal.pone.0265087 (PMC8912191; doi:10.1371/journal.pone.0265087)
Supplement: S6 File — (PDF) [file pone.0265087.s006.pdf]

Dear Expert:

In order to study the relationship between factors influencing very short durations under conditions of resource sufficiency. This interview gives 17 influencing factors, as shown in Table 1. Please score in Table 2 according to Likert five-point scale method, 0 for no effect, 1 for low effect, 2 for medium effect, 3 for high effect, 4 for very high effect. The greater the score, the greater the influence relationship.

Thank you for taking the time to participate in this interview!

Research Group on extremely short construction period

Table 1 Influencing factors of the minimum construction period under sufficient resources.

Constraint Type	influencing factors
project itself	Total floor area（S1）
	Total number of floors（S2）
	Function（S3）
	Structure type（S4）
management	The management level of the owner（S5）
	Contractor management level（S6）
	Designer's capability level（S7）
	Competence level of consultants（S8）
	Management level of the supplier（S9）
logic	Construction technology（S10）
	Maximum construction work surface（S11）
environment	Political environment（S12）
	Natural environment（S13）
	Social environment（S14）
organization	Articulation of materials or devices（S15）
	The connection of construction process steps（S16）
	Construction safety organization（S17）

Table 2 Scoring table of factor correlation

Si	S1	S2	S3	S4	S5	S6	S7	S8	S9	S10	S11	S12	S13	S14	S15	S16	S17
S1	0																
S2		0															
S3			0														
S4				0													
S5					0												
S6						0											
S7							0										
S8								0									
S9									0								
S10										0							
S11											0						
S12												0					
S13													0				
S14														0			
S15															0		
S16																0	
S17																	0
